# Chemical Constituents and α-Glucosidase Inhibitory, Antioxidant and Hepatoprotective Activities of *Ampelopsis grossedentata*

**DOI:** 10.3390/molecules28247956

**Published:** 2023-12-05

**Authors:** Qu-Jing Luo, Wen-Chao Zhou, Xin-Yi Liu, Ya-Jie Li, Qing-Ling Xie, Bin Wang, Chao Liu, Wen-Mao Wang, Wei Wang, Xu-Dong Zhou

**Affiliations:** 1 TCM and Ethnomedicine Innovation & Development International Laboratory, School of Pharmacy, Hunan University of Chinese Medicine, Changsha 410208, China; ishtar61@163.com (Q.-J.L.); zhouwenchao6815@163.com (W.-C.Z.); 18182086610@163.com (X.-Y.L.); 17872355957@163.com (Y.-J.L.); xieql12@126.com (Q.-L.X.); 004146@hnucm.edu.cn (B.W.); 18674403333@163.com (C.L.); 13907442657@163.com (W.-M.W.); 2 Zhangjiajie Meicha Technology Research Center, Hunan Qiankun Biotechnology Co., Ltd., Zhangjiajie 427099, China

**Keywords:** *Ampelopsis*, *Ampelopsis grossedentata*, flavonoids, α-glucosidase inhibitory, antioxidant, hepatoprotective activity

## Abstract

*Ampelopsis grossedentata* is a valuable medicinal and edible plant, which is often used as a traditional tea by the Tujia people in China. *A. grossedentata* has numerous biological activities and is now widely used in the pharmaceutical and food industries. In this study, two new flavonoids (**1**–**2**) and seventeen known compounds (**3**–**19**) were isolated and identified from the dried stems and leaves of *A. grossedentata*. These isolated compounds were characterized by various spectroscopic data including mass spectrometry and nuclear magnetic resonance spectroscopy. All isolates were assessed for their α-glucosidase inhibitory, antioxidant, and hepatoprotective activities, and their structure–activity relationships were further discussed. The results indicated that compound **1** exhibited effective inhibitory activity against α-glucosidase, with an IC_50_ value of 0.21 μM. In addition, compounds **1**–**2** demonstrated not only potent antioxidant activities but also superior hepatoprotective properties. The findings of this study could serve as a reference for the development of *A. grossedentata-derived* products or drugs aimed at realizing their antidiabetic, antioxidant, and hepatoprotective functions.

## 1. Introduction

*Ampelopsis grossedentata*, locally called “Meicha” or “Tocha”, is a species of perennial woody vine, mainly distributed in southern China. In folk, *A. grossedentata* is a valuable medicinal and edible plant and is always used as a traditional herbal tea which is made from its leaves and tender stem. As a medicinal plant, *A. grossedentata* has been used for centuries for various therapeutic purposes, to prevent and treat symptoms such as colds, fevers, sore throats, and toothaches [1,2]. This plant is known as a rich source of flavonoids, especially dihydromyricetin. Previous phytochemical study of *A. grossedentata* mainly includes flavonoids, polyphenols, steroids, terpenes, and volatile components [1,2]. Furthermore, it has been discovered to have a wide range of biological activities, including antioxidant [3,4], liver protection [5,6], antidiabetic [7,8], antitumor [9,10], antiviral [11], anti-inflammatory [12], and antimicrobial [13] effects.

**Figure 1:**
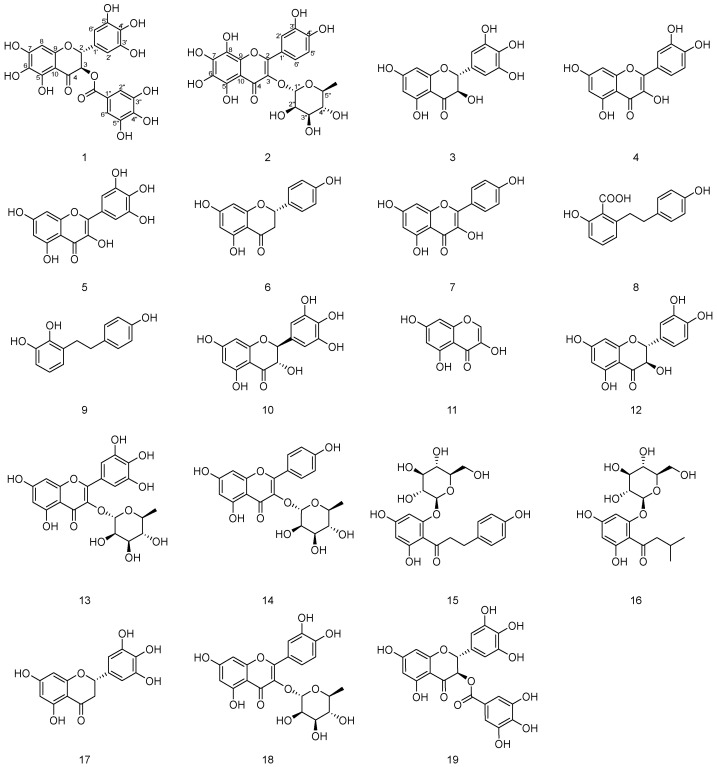
Structures of isolated compounds from the leaves of *A. grossedentata*.

Currently, *A. grossedentata* gets increasing attention due to its long history of use and diverse bioactivities. It has become popular through its use in dietary supplements and other functional products because of its great taste and abundant health benefits. Although about 40 compounds have been found in it, its biological activity has so far been attributed to its most abundant flavonoid, dihydromyricetin. Due to our efforts to search for natural products with novel structures and various biological activity [14,15], nineteen compounds (**1**–**19**), including two new flavonoids named meichasu A–B (**1**–**2**) and seventeen other known compounds, were isolated from the alcoholic extract of *A. grossedentata* leaves in the present work (Figure 1). Among them, nine compounds were isolated from *A. grossedentata* for the first time. Moreover, the biological properties of all the isolates were investigated, revealing α-glucosidase inhibitory, antioxidant, and hepatoprotective capacities. The results indicated that compound **1** exhibited potent inhibitory activity against α-glucosidase, and compounds **1**–**2** showed stronger antioxidant activities than the positive control ascorbic acid. Herein, the details of the isolation, structure identification, and bioactivity of these compounds are presented.

## 2. Results and Discussion

### 2.1. Structure Elucidation

**Figure 2:**
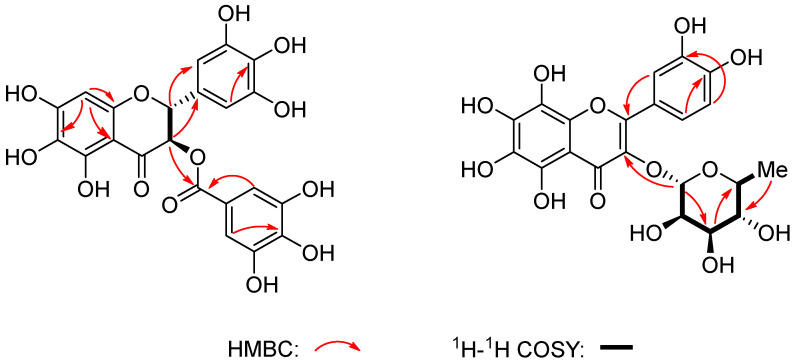
Key HMBC and ^1^H-^1^H COSY correlations of compounds **1**–**2**.

Meichasu A (compound **1**) was obtained as an amorphous gum. The molecular formula C_22_H_16_O_13_ with 15 degrees of unsaturation (Figure 2) was indicated by the [M + H]^+^ peak at *m*/*z* 489.0664 (calcd for C_22_H_17_O_13_^+^, 489.0664) in the HR-ESI-MS and supported by the ^13^C-NMR data (Table 1). IR absorptions at 3388 cm^−1^ and 1636 cm^−1^ suggested the presence of hydroxyl and carbonyl groups. The ^1^H-NMR spectrum of compound **1** exhibited the presence of 1,2,3,5-tetrasubstituted aromatic rings, showing two sets of two-proton singlets [*δ*_H_6.97 (2 H, s); *δ*_H_6.52 (2 H, s)], which were attributed to pyrogallol moiety together with an aromatic proton at *δ*_H_5.96 (1H, s). Meanwhile, a vicinal coupling system of two methines was indicated by the signals at *δ*_H_5.84 (d, *J* = 11.4 Hz) and 5.31 (d, *J* = 11.4 Hz). The ^13^C NMR and DEPT spectrum of compound **1** exhibited 18 carbon resonances corresponding to the above portions and seven quaternary carbons including two carbonyl carbons (*δ*_C_193.4, 166.8) and five aromatic quaternary carbons (Table 1). These spectroscopic data indicated that compound **1** belonged to the number of compounds with flavanonol skeleton similar to compound **19**. A comparison of their data suggested that the difference between them is one additional hydroxyl group at C-6 in compound **1**, which was further confirmed by a detailed analysis of the 2D NMR spectra (Figure 2). In addition, the galloyl group was proved to be linked at C-3 from the HMBC cross-peak of H-3 with its carbonyl at *δ*_C_166.8.

The stereochemistry of C-2 and C-3 in compound **1**, was determined by the coupling constant and the electronic circular dichroism (CD) spectra. The large coupling constant (*J* = 11.4 Hz) between H-2 and H-3 in ^1^H NMR spectrum also revealed they were *trans*-oriented (see the Supplementary Materials). Meanwhile, the positive Cotton effect at 300–340 nm indicated a 2*R* configuration. Taking the above into account, the absolute configuration at C-2 and C-3 could be determined to be 2*R*, 3*R*. Thus, the structure of compound **1** was established as shown above.

Meichasu B (compound **2**) was assigned a molecular formula of C_21_H_20_O_13_ determined on the basis of its positive HRESIMS [M + NH_4_] ^+^ ion peak at *m*/*z* 498.1209 (calcd 498.1242) and the negative ion peak [M − H]^−^ at *m*/*z* 479.0851 (calcd 479.0831). The IR spectrum showed absorption bands for hydroxyl (3364 cm^−1^) and carbonyl (1651 cm^−1^) groups. Detailed analysis for 1D and 2D NMR data (Table 1) indicated that compound **2** possessed a flavonol skeleton with an ABX coupling system in B ring [*δ*_H_7.33, (1 H, d, 2.1 Hz), 7.31 (1 H, dd, 8.3, 2.1 Hz), 6.91 (1 H, d, 8.3, Hz)]. In addition, the presence of a rharmnosyl moiety was evidenced by the characteristic signals (*δ*_C_103.5, 73.3, 72.1,72.0, 71.9, 17.7) combined with the ^1^H-^1^H COSY and HMBC spectra. These NMR data above implied the similarity of compound **2** to the known flavonol, glycoside quercitrin (compound **18**) [16], except for the presence of two additional hydroxyls at C-6 and C-8. Their chemical shifts were determined by comparing the ^13^C NMR data with those of quercitrin and the result of modeling in ChemDraw software. Moreover, in the HMBC spectra, the cross-peak between *δ*_H_5.35 (H-1′) and *δ*_C_136.2 (C-3) demonstrated the rharmnosyl group was attached at C-3 (Figure 2). The coupling constant (*J* = 1.7 Hz) of the anomeric proton indicated a *α*-rharmnosyl moiety. Thus, the structure of compound **2** was determined as shown.

In addition, the structures of the known compounds were identified as (2*R*,3*R*)-Dihydromyricetin (**3**) [17], Quercetin (**4**) [18], Myricetin (**5**) [19], Naringenin (**6**) [20], Kaempferol (**7**) [18], lunularic acid (**8**) [21], lunularin (**9**) [22], (2*S*,3*S*)-Dihydromyricetin (**10**) [23], 3,5,7-Trihydroxychromone (**11**) [24], Taxifolin (**12**) [25], Myricitrin (**13**) [18], Afzelin (**14**) [26], Phloridzin (**15**) [27], 2-O-[1-(3-Methylbutyryl) phloroglucin-ol]-β-D-glucopyranoside (**16**) [28], 5,7,3′,4′,5′-Pentahydroxy flavanone (**17**) [29], Quercitrin (**18**) [16], and (2*R*,3*R*)-3,4-Dihydro-5,7-dihydroxy-4-oxo-2-(3,4,5-trihydroxyphenyl)-2H-1-benzopyran-3-yl 3,4,5-trihydroxybenzoate (**19**) [30] by comparing their spectroscopic data with those reported in the literature.

### 2.2. α-Glucosidase Inhibition Assay

The inhibitory effects of compounds **1**–**19** against α-glucosidase was assessed by determining their IC_50_ values, with comparison of that of the positive control acarbose. The compounds with lower IC_50_ values exhibited greater enzymatic inhibition. As a result (Table 2), compounds **1**, **4**, **5**, **10**, and **19** showed strong α-glucosidase inhibitory activity with IC_50_ values ranging from 0.21 to 1.88 μΜ, which were mainly concerned with two types of skeletons: flavonols and dihydroflavonols. Among them, these dihydroflavonols (**1**, **10**, and **19**) showed better effects than the flavonols (**4**, **5**). After them, compounds **7**, **8**, **9**, **11**, **16**, and **17** showed good inhibitory activities against α-glucosidase with IC_50_ values between 2.90 and 10.91 μM. In addition, the other compounds showed weak activities with less than 50% inhibition of α-glucosidase at a concentration of 20 μM. 

Comparing the dihyroflavonols (**1**, **3**, **10**, **12**, and **19**), one can observe that only compounds **1** and **19**, which have a galloyl substituent at the C-3 position, exhibited strong α-glucosidase inhibitory activity with IC_50_ values of 0.21 and 1.59 μM, respectively. At the same time, the weak inhibitory activity of compound **3**, which has no galloyl substituent at all, indicates that the presence of a galloyl substituent at the C-3 position can greatly enhance the inhibitory activity against α-glucosidase, which verified the previous findings [31]. Although compounds **3** and **10** were optical isomers to each other, compound **10** exhibited significantly stronger inhibitory activity against α-glucosidase, which may indicate that compound **10** has a better steric configuration to bind more effectively to the active site of α-glucosidase than compound **3**. According to the order of the activities of these flavonols (compounds **4**, **5,** and **7**), showing **4** > **5** > **7**, the number of hydroxyl groups in B ring may also be an important factor enhancing α-glucosidase inhibition activity.

Compounds **13**, **14**, **15,** and **18** at all inhibited α-glucosidase, minimally or not, which was in line with previous research that attached glycosyl groups to the C-3 position within ring C, reducing the potency to inhibit α-glucosidase [32]. 

### 2.3. Antioxidant Effect

Three methods, DPPH, ABTS, and FRAP assay, were used to evaluate antioxidant activities of all the isolates in vitro, and the results are shown in Table 3. The results of these three experiments showed that compounds **1**–**5**, **10**, **12**, **13,** and **17**–**19** exhibited stronger antioxidant activity than the positive drug, ascorbic acid, and the activity of compound **7** was similar to that of ascorbic acid. The other compounds had weaker or no significant antioxidant activities. This finding once again confirms that flavonoid compounds have good antioxidant activities. In the DPPH assay, compound **19** exhibited the strongest antioxidant activity with an IC_50_ value of 17.31 μM, while in the ABTS assay, compound **4** showed the strongest activity with an IC_50_ value of 2.76 μM. In contrast to the DPPH and ABTS assays, in the FRAP assay, the greater the concentration of ferrous ions, the better the antioxidant activity of these isolates. Compound **19** showed the strongest activity with a FRAP value of 2.79 μmol/mL. 

The new compounds **1** and **2** both exhibited strong antioxidant activities. Compound **1** was more active than compound **2** in DPPH and ABTS assays, whereas the opposite result was observed in FRAP assays, which may be due to different mechanisms of each reaction. Comparing the results of flavonols (**4**, **5,** and **7**) and flavonol glycosides (**2**, **13**, **14**, and **18**), the flavonols showed roughly higher antioxidant activity than the latter. The order of the activities of the flavonol glycosides with one single rhamnose was **2** > **13** > **18** > **14**, suggesting that the more hydroxyl groups there are, the better the activity is, which is consistent with previous findings [33]. The above comparison also showed that the activities of these glycosides were weaker than that of aglycones, which indicated the presence of sugar groups may decrease the antioxidant activity of flavonoids. Considering the isomers of compounds **3** and **10**, the results showed that the activity of compound **3** was stronger than those of compound **10**, indicating that the configuration of compound **3** was more advantageous. In addition, the comparison of the activities of these three compounds (**1**, **3,** and **19**) implied the presence of galloyl was vital for the effects, and the presence of extra hydroxyl in the A-ring seemed not necessarily to increase the activities. 

### 2.4. Hepatoprotective Activity

**Figure 3:**
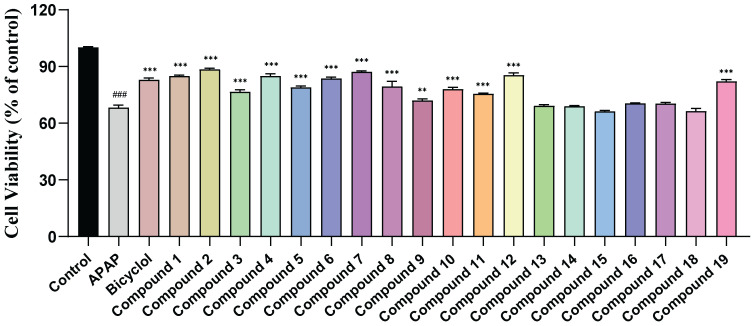
Hepatoprotective activity of compounds **1**–**19** (20 μM). Note: n = 3, mean ± SD. Compared with the control: ### *p* < 0.001; compared with the APAP: ** *p* < 0.01 and *** *p* < 0.001.

Since previous studies have shown that *A. grossedentata* displayed good properties in liver protection [5,6], an APAP-induced HepG2 cell injury model was established to evaluate the protective effect of the isolated compounds at a concentration of 20 μM on HepG2 cells, and the hepatoprotective drug bicyclol was used as a positive control. The results (Figure 3) demonstrated that compounds **1**, **2**, **4**, **6**, **7**, and **12** displayed significant protective effect against acetaminophen-induced HepG2 cell injury, with a corresponding increase in cell viability from 68.24 ± 1.34% to 84.93 ± 0.58%, 88.46 ± 0.63%, 84.90 ± 1.21%, 83.7 ± 0.67%, 87.08 ± 0.60%, and 85.43 ± 1.22%. All these parameters were higher than in the preparation of cell viability positive control of 83.01 ± 0.82%. Compounds **3**, **5**, **8**–**11,** and **13** also exhibited moderate hepatoprotective activities, while compounds **13**–**18** did not show an obvious liver protection effect. These results again demonstrate the superior hepatoprotective activity of flavonoids.

The flavonols (compounds **4**, **5,** and **7**) all exhibited strong hepatoprotective activity, while their corresponding flavonol glycosides (compounds **13**, **14,** and **18**) were essentially inactive. This result indicated that the presence of glycosyl at C-3 in flavonols can significantly reduce their hepatoprotective activities. Furthermore, compound **19**, which is a galloyl derivative of compound **3** by C-3, exhibited stronger activity than compound **3**, indicating that the presence of galloyl may enhance the hepatoprotective activity.

Among all the compounds, compound **2** was the most effective in increasing the survival rate of HepG2 cells. Compared with the inactive compound **18**, the difference between the two was that compound **2** had two additional hydroxyl groups on its A-ring. In addition, compound **1** exhibited better activity compared to compound **19**, despite the fact that the only structural difference between the two compounds is that compound **1** has an extra hydroxyl group on its A-ring. These results suggested that the number of hydroxyl groups on the A-ring appears to affect the activities. Furthermore, by comparing compounds **4**–**5** with **7**, **3** with **12**, **6** with **17**, we also found that the number of hydroxyls on the B-ring has a significant impact on the hepatoprotective activity.

## 3. Materials and Methods

### 3.1. General Experimental Procedures

Optical rotations were measured with a Rudolf AUTOPOL III polarimeter (USA). Circular dichroism (CD) spectra were recorded on a JASCO J-1500-150 spectropolarimeter. UV spectra (methanol) were measured with a PerkinElmer Lambda 650 spectrophotometer. IR spectra were obtained on a PerkinElmer Frontier MIR spectrophotometer. In addition, 1D and 2D NMR spectra were recorded on Bruker Avance-600 NMR spectrometer with TMS as internal standard. Unless stated otherwise, all chemical shifts (δ) are reported in ppm relative to the solvent signals, and coupling constants are reported in Hertz. HR-ESIMS data were acquired on a Waters Xevo G2-S QTof mass spectrometer. 

Semi-preparative HPLC was conducted on an Agilent 1260 Infinity II HPLC system with an Agilent Pursuit XRs 10 C18 column (250 × 10 mm, 5 μm). Column chromatography (CC) was performed with silica gel (100–200 and 200–300 mesh, Qingdao Marine Chemical Group Co., Shandong, China), ODS RP-18 gel (40–63 mm, Merck, Darmstadt, Germany), Sephadex LH-20 (Pharmacia, Uppsala, Sweden) and Macroporous adsorbent resin AB-8 (0.3–1.25 mm, Tianjin City Guang Fu Tech. Development Co., Ltd., Tianjin, China). Thin-layer chromatography (TLC) was employed to monitor the CC fractions, with visualization achieved through the application of 1% vanillin in H_2_SO_4_ as a spraying reagent.

### 3.2. Chemicals and Reagents

DPPH (2,2-diphenyl-1-picrylhydrazyl), ABTS (2,2′-azinobis(3-ethylbenzthiazoline-6-sulphonic acid)), TPTZ (2,4,6-tris(2-pyridyl)-S-triazine), and ascorbic acid were obtained from Beijing Solarbio Science & Technology Co., Ltd (Beijing, China). α-Glucosidase was purchased from Shanghai Yuanye Biotechnology Co., Ltd (Shanghai, China). p-NPG(p-nitrophenyl-α-D-glucopyranoside), acarbose, acetaminophen, bicyclol, anhydrous sodium carbonate, ferric chloride, and potassium persulfate were purchased from Shanghai Aladdin Biochemical Technology Co., Ltd. (Shanghai, China). Methanol and acetonitrile (HPLC-grade) were purchased from Sigma-Aldrich (Wuxi) Life Science & Tech. Co., Ltd. (Wuxi, China). Purified water was purchased from China Resources C’estbon Beverage (China) Co., Ltd. (Changsha, China). Anhydrous ethanol, dimethyl sulfoxide (DMSO), and all other chemicals of analytical grade were purchased from Sinopharm Chemical Reagent Co., Ltd. (Shanghai, China).

### 3.3. Plant Material

The dried leaves of *A. grossedentata* were collected from Zhangjiajie (29°20′13″ N, 110°18′52″ E), Hunan Province, China, in July 2020, and identified by associate professors, Xu-Dong Zhou (Hunan University of Chinese Medicine, Changsha, China) and Wen-Mao Wang (Zhangjiajie Meicha Technology Research Center, Zhangjiajie, China). The samples were stored in the TCM and Ethnomedicine Innovation & Development International Laboratory, Hunan University of Chinese Medicine, Hunan Province.

### 3.4. Extraction and Isolation

The extract (1.0 kg, 21.3% yield) from leaves and stems of *A. grossedentata* (4.7 kg) was provided by Zhangjiajie Meicha Technology Research Center of Hunan Qiankun Biotechnology Co., Ltd. (Zhangjiajie, China), which was obtained by reflux extraction with 95% ethanol. Firstly, the extract was dissolved in 90% ethanol and left at room temperature (25 °C) to recrystallize to obtain the main constituent dihydromyricetin (compound **3** with large amounts). Then, a large amount of dihydromyricetin crystals were removed by suction filtration and then washed repeatedly with distilled water (3 × 50 mL) 3 times to obtain filtrate. Secondly, the filtrate was concentrated until there was no ethanol in it, redissolved with a small amount of water, and then added to the macroporous adsorption resin (AB-8) CC for separation. The volume ratio of extract and macroporous adsorbent resin was 1:10, and 15 fractions (Fr.1-Fr.15) were obtained by gradient elution of H_2_O-EtOH (100:0 → 5:95, *v*/*v*).

Fr.9 was dissolved in methanol and separated by Sephadex LH-20 CC (eluting with MeOH) to obtain Fr.9-1-Fr.9-14. A part of Fr.9-9 was then purified by semi-preparative HPLC (MeOH-H_2_O, 50:50, 3 mL/min, 9.7 min) to yield compound **1** (5.3 mg). The other part was repeatedly eluted twice with Sephadex LH-20 CC eluting with MeOH, to obtain compound **10** (4.2 mg). Compounds **11** (2.6 mg) and **12** (27.3 mg) were obtained by elution of Fr.9-5 on silica gel column with CH_2_Cl_2_-EtOAc (100:0 → 20:80, *v*/*v*). The Fr.9-5 was then separated using a semi-preparative HPLC (MeOH-H_2_O, 50:50, 3 mL/min, 8.6 min) to give compounds **2** (10.1 mg) and **13** (16.7 mg). Fr.9-3 was separated by Sephadex LH-20 CC eluting with MeOH, and then further eluted by silica gel column chromatography (DCM-EtOAc, 2:1 → 1:10, *v*/*v*) to yield compound **15** (14.5 mg). 

Fr.13 was chromatographed by Sephadex LH-20 (MeOH) to obtain Fr.13-1-Fr.13-17, and then Fr.13-12, Fr.13-13, Fr.13-9, Fr.13-11 were purified by Sephadex LH-20 (MeOH), respectively. Thus, compounds **4** (26.7 mg), **5** (24.8 mg), **6** (4.5 mg), and **7** (5.0 mg) were obtained. Fr.13-6 was separated by Sephadex LH-20 in MeOH and then purified by semi-prepared HPLC (MeOH-H_2_O, 54:46, 3 mL/min) to afford a mixture of compounds **8** and **9**. The mixture was further purified by Sephadex LH-20 in MeOH and then compounds **8** (2.3 mg) and **9** (1.4 mg) were obtained, separately.

Fr.12 was subjected to Sephadex LH-20 (MeOH) to obtain Fr.12-1-Fr.12-19. Fr.12-7 was purified by Sephadex LH-20 (MeOH) and then separated by semi-preparate HPLC (MeOH-H_2_O, 52:48, 3 mL/min) to yield compound **14** (3.4 mg). Fr.12-4 was purified by silica gel column chromatography (DCM-EtOAc, 40:1 → 1:5, *v*/*v*) to yield compound **16** (5.7 mg). Fr.12-12 was subjected to silica gel CC (DCM-EtOAc, 50:1 → 1:1, *v*/*v*) to obtain compound **17** (3.1 mg). Fr.12-8 was separated by silica gel column chromatography (DCM-EtOAc, 50:1 → 1:10, *v*/*v*) and further purified by semi-preparative HPLC (MeOH-H2O, 60:40, 3 mL/min) to obtain compound **18** (23.0 mg). Fr.12-15 was eluted twice by silica gel CC (DCM-EtOAc, 20:1 → 1:5, *v*/*v*), and then isolated by semi-preparative HPLC (MeOH-H_2_O, 55:45, 3 mL/min) to obtain compound **19** (38.8 mg).

Meichasu A (**1**): yellow, amorphous gum; ${\left[\alpha \right]}_{D}^{25}$ + 14.03 (*c* 0.05, MeOH); UV (MeOH) λ_max_ (log *ε*): 210 (2.86), 293 (2.93) nm; CD (MeOH) λ_max_ (Δ*ε*): 280 (−0.2), 330 (+0.8) nm; IR (KBr) *ν*_max_ 3388, 2925, 1636, 1210, 1034 cm^−1^; ^1^H and ^13^C NMR data (CD_3_OD, 600/151 MHz), see Table 1; HRESIMS *m*/*z* 489.0664 [M + H]^+^ (calcd for C_22_H_17_O_13_^+^, 489.0664).

Meichasu B (**2**): yellow, amorphous gum; ${\left[\alpha \right]}_{D}^{25}$ − 147.1 (*c* 0.15, MeOH); UV (MeOH) λ_max_ (log *ε*): 204 (2.86), 256 (2.91), 350 (2.97) nm; IR (KBr) *ν*_max_ 3364, 1651, 1201, 1089 cm^−1^; ^1^H and ^13^C NMR data (CD_3_OD, 600/151 MHz), see Table 1; HRESIMS *m/z* 498.1209 [M + NH_4_]^+^ (calcd for C_21_H_24_NO_13_^+^, 498.1242) and *m*/*z* 479.0851 [M − H]^−^ (calcd for C_21_H_19_O_13_^−^, 479.0831).

### 3.5. α-Glucosidase Inhibitory Assay

The α-glucosidase inhibitory activities of isolated compounds were evaluated by using a method previously described, but with minor modifications [34]. The experiment was conducted using a 96-well plate, and each reaction had a total volume of 200 μL. All isolated compounds and acarbose were dissolved and diluted in DMSO (Dimethyl sulfoxide), p-NPG (p-nitrophenyl-α-D-glucopyranoside) and enzymes were dissolved in PBS (Phosphate-buffered saline). Briefly, 98 μL of PBS (0.1 M, pH 6.8) was added to each well. Then, 2 μL of different concentrations of sample solutions and 25 μL of α-glucosidase solution (0.25 U/mL) were added. After shaking and mixing slightly, the 96-well plate was placed in a 37 °C constant-temperature incubator and incubated for 20 min. Next, 25 μL of p-NPG (4 mM) was added to the plate to initiate the reaction, and it was further incubated in a 37 °C constant-temperature incubator for 15 min. Then, 50 μL of Na_2_CO_3_ (0.2 M) solution was quickly added to each well to terminate the reaction. The absorbance was measured at 405 nm using a microplate reader, and the results were obtained from a minimum of three independent experiments. Acarbose was used as positive control. The experiment was divided into 4 groups: group A was the sample group containing enzyme, group B replaced the enzyme with PBS, group C replaced the sample or acarbose with DMSO, and group D was the blank control group without enzyme and sample, and other reagents were consistent with group A.

The percentage inhibition rate (%) of α-glucosidase for each test sample was calculated as:

Inhibition rate (%) = [1 − (OD_A_ − OD_B_)/(OD_C_ − OD_D_)] × 100%

The IC_50_ values were calculated by using GraphPad Prism 9.0 software.

### 3.6. Measurement of Antioxidant Activity

#### 3.6.1. DPPH Assay

The DPPH free radical scavenging capacity of isolated compounds was determined according to a modified method [35]. The experiment was conducted using a 96-well plate, and each well had a total volume of 200 μL. All isolated compounds and positive drug were dissolved and diluted in DMSO and DPPH (2,2-Diphenyl-1-picrylhydrazyl) was dissolved in anhydrous ethanol. Briefly, 190 μL of a working solution of DPPH (0.2 mM) was added to each well. Then, 10 μL of a sample solution of different concentrations (1.6, 1.2, 0.8, 0.4, 0.2, and 0.1 mM) was added, mixed well, and left to incubate in the dark at room temperature for 60 min. After that, the absorbance values of each well were measured at 517 nm using a microplate reader and the results were obtained from a minimum of three independent experiments. Ascorbic acid was used as the positive control. DMSO was used to replace the sample solution as a blank and absolute ethanol was used to replace the DPPH solution as a control.

The percentage free radical scavenging rate (%) of each test sample for DPPH was calculated as:

Free radical scavenging rate (%) = [A_blank_ − (A_sample_ − A_contral_)] ÷ A_blank_ × 100%

#### 3.6.2. ABTS Assay

The ABTS free radical scavenging capacity of all isolated compounds was measured, the procedure followed a method with minor modifications [36]. The experiment was conducted using a 96-well plate, and each well had a total volume of 200 μL. Equal volumes of ABTS (2,2′-azinobis(3-ethylbenzthiazoline-6-sulphonic acid)) solution (7 mM) and potassium persulfate solution (2.45 mM) were mixed evenly and left to react at room temperature for 12 h in the dark to obtain ABTS radical cation. The mixture was then diluted with anhydrous methanol to achieve an absorbance value of about 0.7 ± 0.02 units at 405 nm. Then, 190 μL of ABTS working solution was added to each well, followed by 10 μL samples of varying concentrations (0.8, 0.4, 0.2, 0.1, 0.05, 0.025, 0.0125, and 0.00625 mM) that were dissolved and diluted with DMSO. After mixing well, the samples were incubated at room temperature for 6 min away from light. The subsequent experimental method is basically consistent with the DPPH assay, the only difference is that ABTS assay detects the absorbance value at 405 nm.

The percentage radical cation scavenging rate (%) of each test sample for ABTS was calculated as follows:

Radical cation scavenging rate (%) = [A_blank_ − (A_sample_ − A_contral_)] ÷ A_blank_ × 100%

The IC_50_ values were calculated by using GraphPad Prism 9.0 software.

#### 3.6.3. FRAP Assay

The total antioxidant capacity of the samples was measured by the FRAP (ferric reducing ability of plasma) assay, with slight modifications [37]. The acetate buffer (0.3 M, pH 3.6), TPTZ (2,4,6-tris(2-pyridyl)-S-triazine) solution (10 mM) in 40 mM HCl and ferric chloride aqueous solution (10 mM) were uniformly mixed at a volume ratio of 7:1:1 to obtain the FRAP working solution. FRAP working solution was preheated for 10 min in a constant temperature incubator at 37 °C before use. The experiment was conducted using a 96-well plate, and each well had a total volume of 204 μL. Then, 180 μL of FRAP working solution was added to each well, followed by 6 μL of sample solution (0.68 mM), and finally 18 μL of distilled water. Then, the mixture was stirred well and left to react at room temperature for 10 min. The absorbance values of each well were measured at 593 nm using a microplate reader and the results were obtained from at least three independent experiments. The sample solution was replaced with DMSO as a blank solution and ascorbic acid was used as a positive control. Different concentrations of FeSO_4_·7H_2_O (0.075–0.00078 μmol/mL) were used to establish the standard curve. The antioxidant capacity of the sample was expressed as the Fe^2+^ concentration (μmol/mL) required to achieve the same absorbance change value. The final result was calculated by GraphPad Prism 9.0 software and presented as mean ± standard deviation (SD) of μmol Fe^2+^ per milliliter.

### 3.7. Hepatoprotective Activity

HepG2 cells were maintained in DMEM medium supplemented with 10% fetal bovine serum, 100 U/mL penicillin G, and 100 μg/mL streptomycin. The cells were seeded in culture flasks and placed in a 5% CO_2_ incubator at 37 °C for routine cultivation. The hepatoprotective activities of the isolated samples were tested according to the modified method [38]. To evaluate the effect of test samples on cell viability, a CCK-8 assay was performed. The experiment was conducted in 96-well plates, and 100 μL of HepG2 cells in logarithmic growth phase (8 × 10^5^ cells/mL) were seeded in each well and cultured for 24 h. After that, samples (final concentration of 20 μM) and acetaminophen (APAP, final concentration of 6 mM) were added at one time to incubate for another 48 h. Then, 10 μL CCK-8 was added to each well and incubated for 1 h, followed by measuring the absorbance at a wavelength of 450 nm using a microplate reader.

## 4. Conclusions

In the present study, a comprehensive phytochemical investigation was conducted to isolate 19 compounds from the traditional dietary tea, *A. grossedentata.* Their structures were characterized through extensive spectroscopic analysis (NMR and HR-ESIMS). These included two previously undescribed flavonoids, which were named meichasu A–B (**1**–**2**), and seven compounds that were isolated for the first time from *A. grossedentata*. Based on the traditional applications of the tea and the structural characteristics of these isolates, the α-glucosidase inhibitory, antioxidant, and hepatoprotective activities of the isolated compounds were evaluated. The results showed that compound **1** exhibited potent inhibitory activity against α-glucosidase, with an IC_50_ value of 0.21 μM. The antioxidant and hepatoprotective abilities of compounds **1**–**2** were found to be stronger than those of the positive control drug, indicating their broad biological activities. 

*A. grossedentata*, as a traditional herb, is a precious and rich resource for discovering bioactive natural products. These research findings have enriched the knowledge of the chemical diversity of *A. grossedentata*, reflecting the potential α-glucosidase inhibitory, antioxidant, and hepatoprotective effects of the flavonoids contained in this herbal tea, and that these active compounds may have potential therapeutic implications for the treatment of diabetes and liver damages.

## Figures and Tables

**Table 1:** The ^1^H NMR (600 MHz) and ^13^C NMR (151 MHz) data of the samples Meichasu A (**1**) and Meichasu B (**2**) (in CD_3_OD) (δ in ppm, J in Hz).

No.	1		2	
	*δ*_C_	*δ*_H_	*δ*_C_	*δ*_H_
2	82.9	5.31, d, (11.4)	159.3	--
3	74.2	5.84, d, (11.4)	136.2	--
4	193.4	--	179.6	--
5	165.4	--	163.1	--
6	164.2	--	166.3	--
7	169.9	--	166.3	--
8	96.6	5.96, s	158.6	--
9	169.1	--	158.5	--
10	102.1	--	105.8	--
1′	127.9	--	122.9	--
2′	107.8	6.52, s	116.9	7.33, d (2.1)
3′	147.0	--	146.4	--
4′	135.1	--	149.8	--
5′	147.0	--	116.4	6.91, d (8.3)
6″	107.8	6.52, s	122.8	7.31, dd (8.3, 2.1)
1″	120.4	--	103.5	5.35, d (1.7)
2″	110.4	6.97, s	71.9	4.22, dd (3.4, 1.7)
3″	146.4	--	72.1	3.75, dd (9.6, 3.4)
4″	140.2	--	73.2	3.34, t (9.6)
5″	146.4	--	72.0	3.42, dq (9.6, 6.1)
6″	110.4	6.97, s	17.7	0.94, d (6.1)
7″	166.8	--		

**Table 2:** α-glucosidase inhibitory activities of compounds **1**–**19** from *A. grossedentata*.

Compound	IC_50_ ^a^ (μM)	Compound	IC_50_^a^ (μM)
**1**	0.21 ± 0.01	**11**	6.04 ± 0.26
**2**	>20	**12**	>20
**3**	>20	**13**	>20
**4**	1.88 ± 0.08	**14**	>20
**5**	1.83 ± 0.05	**15**	>20
**6**	>20	**16**	10.91 ± 0.51
**7**	2.90 ± 0.12	**17**	4.18 ± 0.11
**8**	10.32 ± 0.31	**18**	>20
**9**	9.42 ± 0.51	**19**	1.59 ± 0.03
**10**	1.69 ± 0.05	Acarbose ^b^	0.06 ± 0.01

^a^ Data were represented as the mean value ± SD, n = 4. Values accompanied by different letters are significantly different (*p* ≤ 0.05). ^b^ Acarbose was employed as the positive control.

**Table 3:** Antioxidant inhibitory activities of compounds **1**–**19** from *A. grossedentata*.

Compound	Antioxidant Activity		
	DPPH Assay IC_50_ ^a^ (μM)	ABTS Assay IC_50_ ^a^ (μM)	FRAP Assay ^a^ (μMOL/ML)
**1**	32.52 ± 0.16	4.43 ± 0.02	1.70 ± 0.00
**2**	44.42 ± 0.63	6.73 ± 0.03	1.96 ± 0.01
**3**	28.26 ± 0.38	5.91 ± 0.11	1.71 ± 0.01
**4**	25.54 ± 0.63	2.76 ± 0.04	2.67 ± 0.01
**5**	29.75 ± 0.59	3.39 ± 0.07	1.72 ± 0.01
**6**	>80	>20	0.07 ± 0.00
**7**	>80	19.91 ± 0.46	0.85 ± 0.01
**8**	>80	>20	0.13 ± 0.01
**9**	>80	>20	0.09 ± 0.00
**10**	43.50 ± 0.36	5.01 ± 0.11	1.43 ± 0.01
**11**	>80	>20	0.15 ± 0.00
**12**	39.21 ± 0.18	7.36 ± 0.08	1.32 ± 0.01
**13**	35.03 ± 0.28	6.88 ± 0.13	1.69 ± 0.02
**14**	>80	>20	0.07 ± 0.00
**15**	>80	>20	0.17 ± 0.01
**16**	>80	>20	0.24 ± 0.01
**17**	43.05 ± 0.94	10.25 ± 0.13	1.18 ± 0.01
**18**	63.51 ± 0.97	9.96 ± 0.16	1.35 ± 0.02
**19**	17.31 ± 0.31	2.19 ± 0.01	2.79 ± 0.01
ascorbic acid ^b^	77.93 ± 0.87	19.01 ± 0.31	0.57 ± 0.01

^a^ Data were represented as the mean value ± SD, n = 4. Values followed by different letters are significantly different (*p* ≤ 0.05). ^b^ Ascorbic acid was employed as the positive control.

## Supplementary Materials

The following supporting information can be downloaded at: https://www.mdpi.com/article/10.3390/molecules28247956/s1, Figure S1. ^1^H NMR spectrum of meichasu A (**1**) (CD_3_OD, 600 MHz).; Figure S2. ^13^C NMR spectrum of meichasu A (**1**) (CD_3_OD, 125 MHz).; Figure S3. DEPT 135° spectrum of meichasu A (**1**) (CD_3_OD, 125 MHz).; Figure S4. HSQC spectrum of meichasu A (**1**).; Figure S5. HMBC spectrum of meichasu A (**1**); Figure S6. ^1^H-^1^H COSY spectrum of meichasu A (**1**).; Figure S7. HR-ESIMS spectrum of meichasu A (**1**).; Figure S8. UV spectrum of meichasu A (**1**).; Figure S9. IR spectrum of meichasu A (**1**).; Figure S10. CD spectrum of meichasu A (**1**).; Figure S11. ^1^H NMR spectrum of meichasu B (**2**) (CD_3_OD, 600 MHz).; Figure S12. ^13^C NMR spectrum of meichasu B (**2**) (CD_3_OD, 125 MHz).; Figure S13. DEPT 135° spectrum of meichasu B (**2**) (CD_3_OD, 125 MHz).; Figure S14. HSQC spectrum of meichasu B (**2**).; Figure S15. HMBC spectrum of meichasu B (**2**).; Figure S16. ^1^H-^1^H COSY spectrum of meichasu B (**2**).; Figure S17. HR-ESIMS spectrum of meichasu B (**2**).; Figure S18. UV spectrum of meichasu B (**2**).; Figure S19. IR spectrum of meichasu B (**2**).
