# Supplementary material for: Chemical Constituents and α-Glucosidase Inhibitory, Antioxidant and Hepatoprotective Activities of Ampelopsis grossedentata
Source: Molecules. 2023 Dec 5;28(24):7956. doi: 10.3390/molecules28247956 (PMC10745659; doi:10.3390/molecules28247956)
Supplement: Supplementary file 1 [file molecules-28-07956-s001.zip › molecules-2735424-supplementary.pdf]

Supporting Information

Chemical Constituents and α-Glucosidase Inhibitory, Antioxidant and Hepatoprotective Activities of Ampelopsis grossedentata

Qu-Jing Luo [1], Wen-Chao Zhou [1], Xin-Yi Liu [1], Ya-Jie Li [1], Qing-Ling Xie [1], Bin Wang [1], Chao Liu [1,2], Wen-Mao Wang [1,2], Wei Wang [1,*] and Xu-Dong Zhou [1,*]

[1] TCM and Ethnomedicine Innovation & Development International Laboratory, School of Pharmacy, Hunan University of Chinese Medicine, Changsha 410208, China; ishtar61@163.com (Q.-J.L.); zhouwenchao6815@163.com (W.-C.Z.); 18182086610@163.com (X.-Y.L.); 17872355957@163.com (Y.-J.L.); xieql12@126.com (Q.-L.X.); 004146@hnucm.edu.cn (B.W.); 18674403333@163.com (C.L.); 13907442657@163.com (W.-M.W.)

[2] Zhangjiajie Meicha Technology Research Center, Hunan Qiankun Biotechnology Co., Ltd., Zhangjiajie 427099, China

* Correspondence: wangwei402@hotmail.com (W.W.); xudongzhou999@hnucm.edu.cn (X.-D.Z.); Tel.: +86-731-8845-8240 (W.W.); +86-731-8845-8240 (X.-D.Z.); Fax: +86-8845-8227 (W.W.); +86-731-8845-8240 (X.-D.Z.)

Contents:

Figure S1. ^{1}H NMR spectrum of meichasu A (**1**) (CD_3OD, 600 MHz).

Figure S2. ^{13}C NMR spectrum of meichasu A (**1**) (CD_3OD, 125 MHz).

Figure S3. DEPT 135° spectrum of meichasu A (**1**) (CD_3OD, 125 MHz).

Figure S4. HSQC spectrum of meichasu A (**1**).

Figure S5. HMBC spectrum of meichasu A (**1**).

Figure S6. ^{1}H-^{1}H COSY spectrum of meichasu A (**1**).

Figure S7. HR-ESIMS spectrum of meichasu A (**1**).

Figure S8. UV spectrum of meichasu A (**1**).

Figure S9. IR spectrum of meichasu A (**1**).

Figure S10. CD spectrum of meichasu A (**1**).

Figure S11. ^{1}H NMR spectrum of meichasu B (**2**) (CD_3OD, 600 MHz).

Figure S12. ^{13}C NMR spectrum of meichasu B (**2**) (CD_3OD, 125 MHz).

Figure S13. DEPT 135° spectrum of meichasu B (**2**) (CD_3OD, 125 MHz).

Figure S14. HSQC spectrum of meichasu B (**2**).

Figure S15. HMBC spectrum of meichasu B (**2**).

Figure S16. ^{1}H-^{1}H COSY spectrum of meichasu B (**2**).

Figure S17. HR-ESIMS spectrum of meichasu B (**2**).

Figure S18. UV spectrum of meichasu B (**2**).

Figure S19. IR spectrum of meichasu B (**2**).

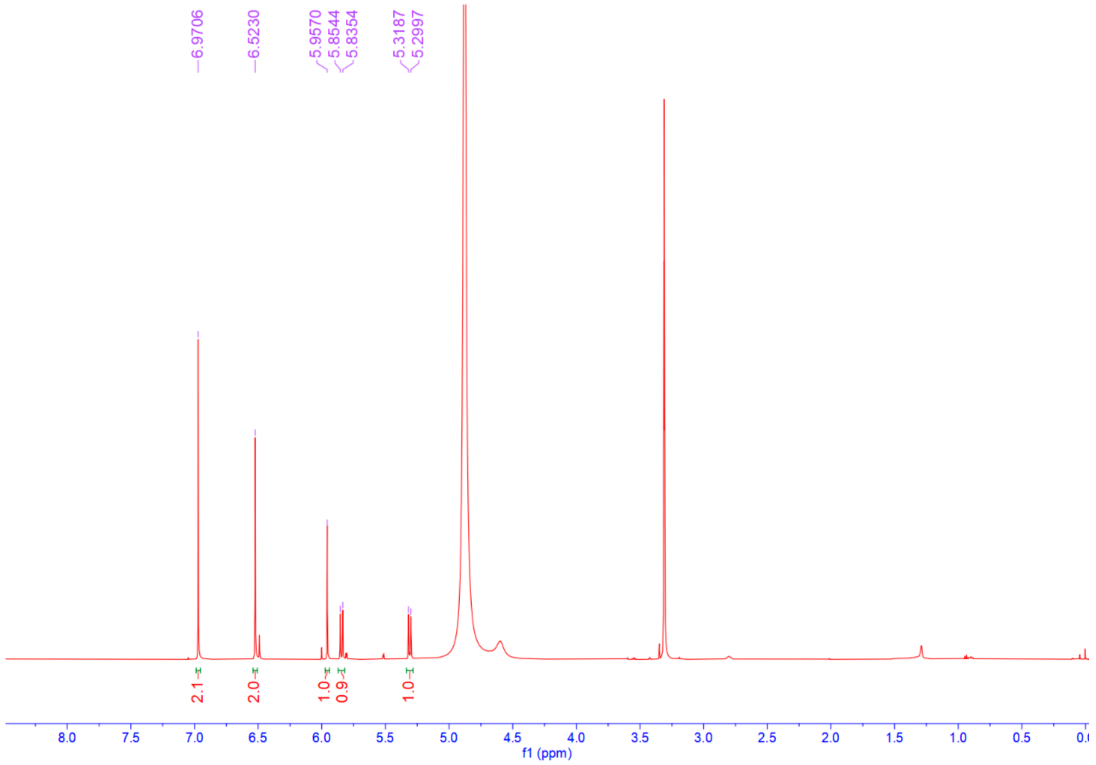

Figure S1. ^{1}H NMR spectrum of meichasu A (**1**) (CD_3OD, 600 MHz).

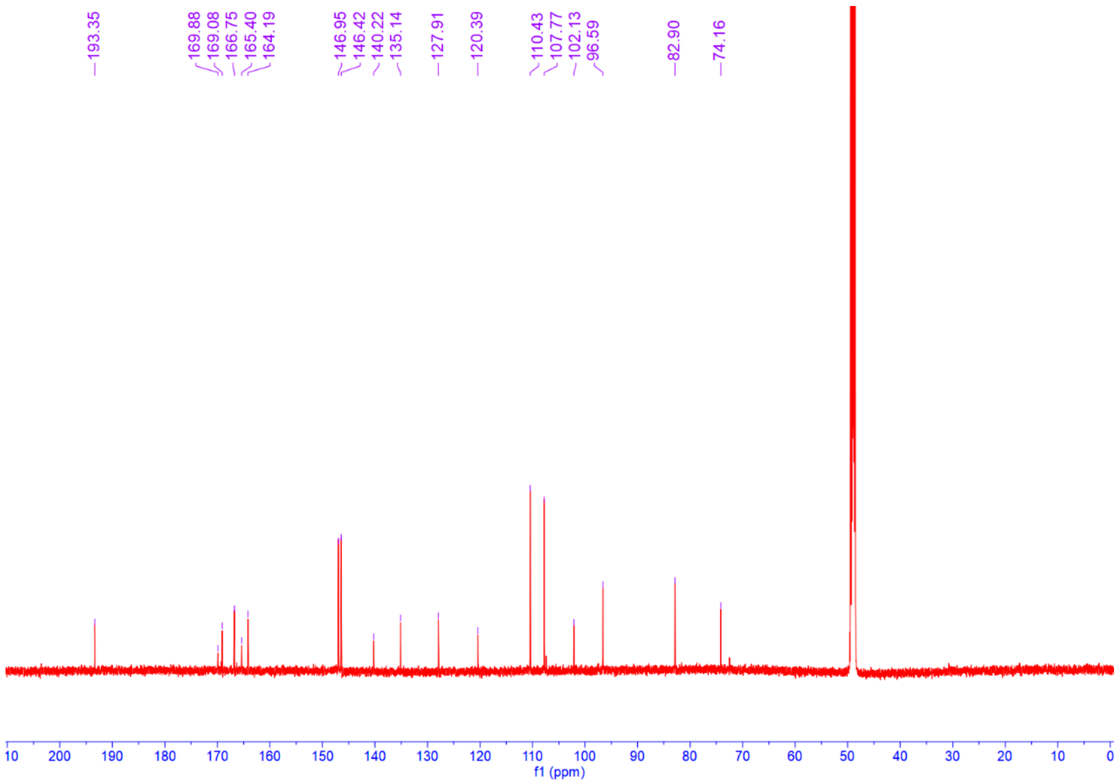

Figure S2. ^{13}C NMR spectrum of meichasu A (**1**) (CD_3OD, 151 MHz).

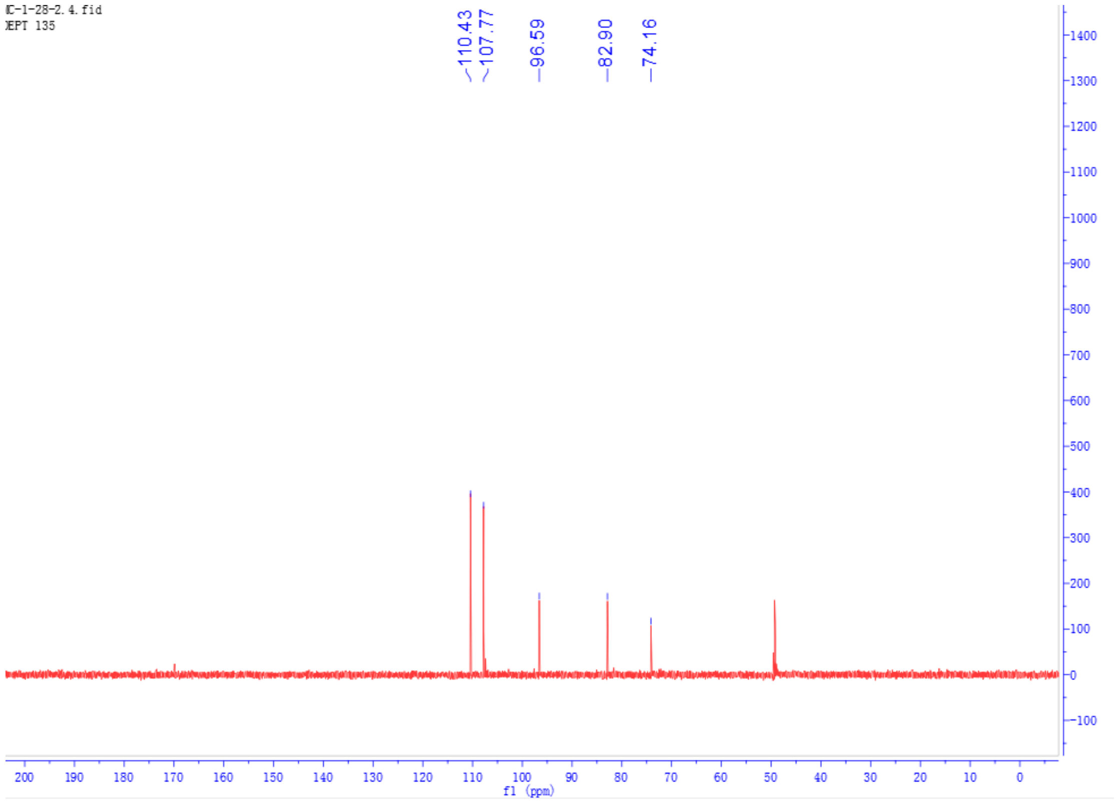

Figure S3. DEPT 135° spectrum of meichasu A (**1**) (CD_3OD, 151 MHz).

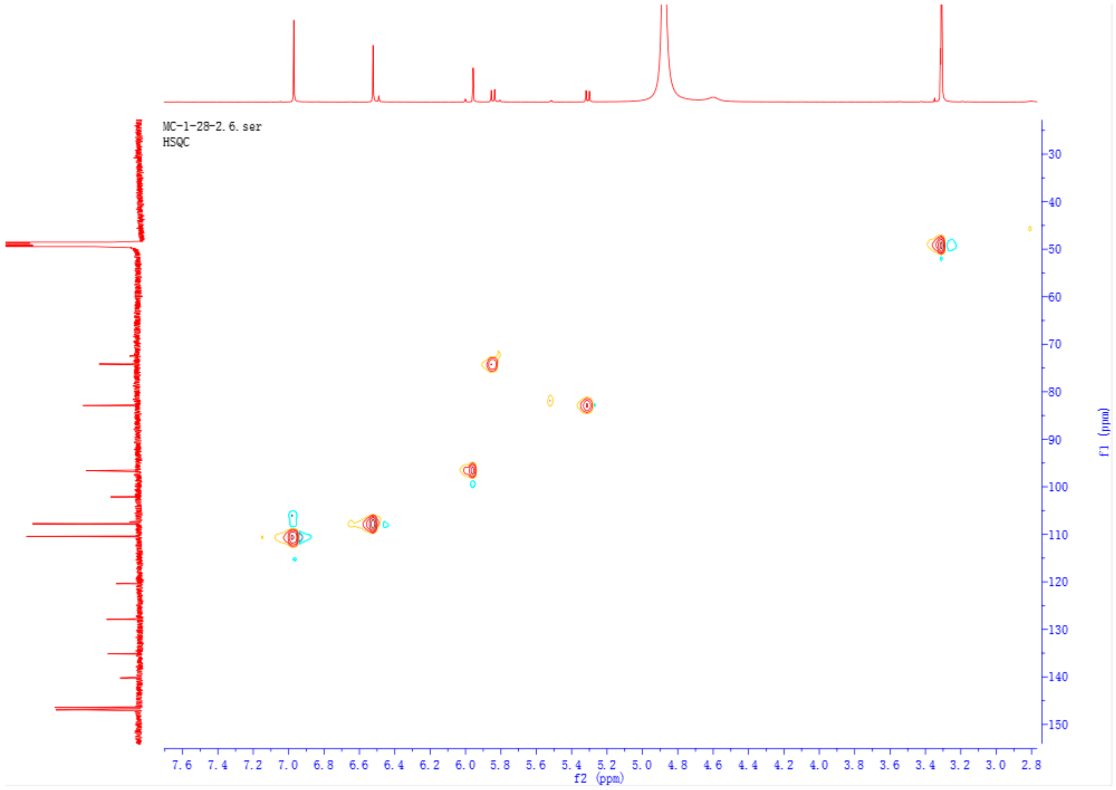

Figure S4. HSQC spectrum of meichasu A (**1**).

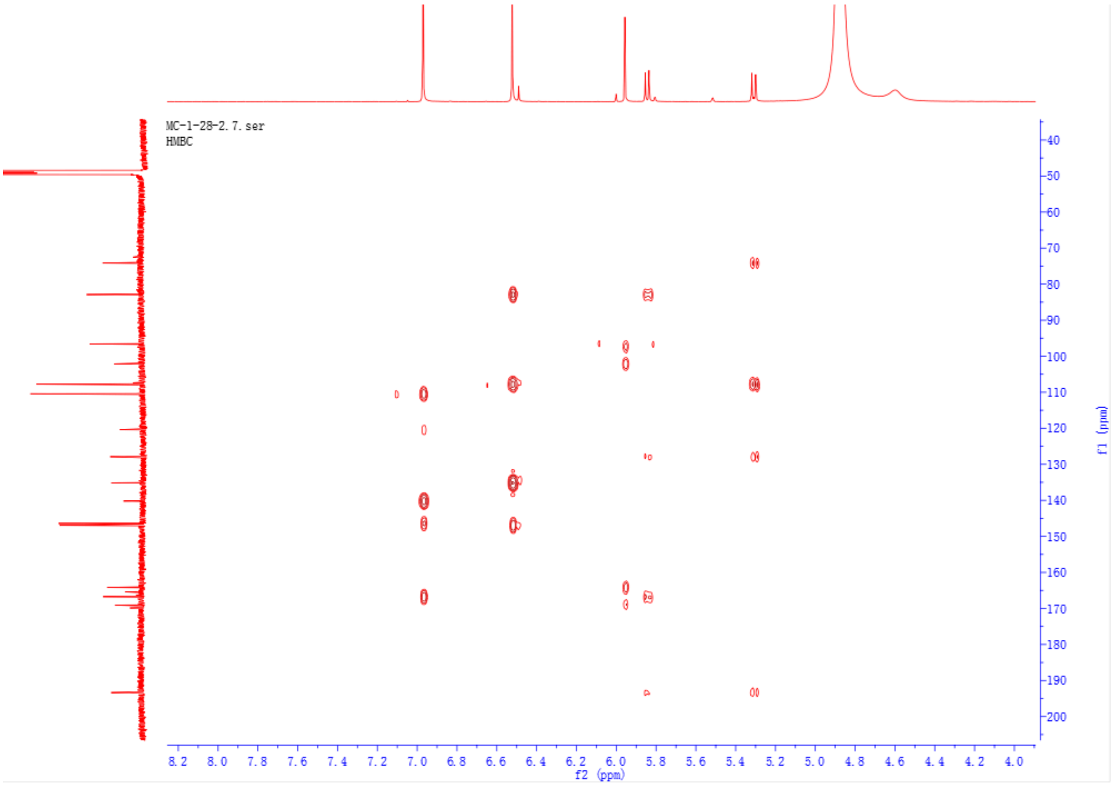

Figure S5. HMBC spectrum of meichasu A (**1**).

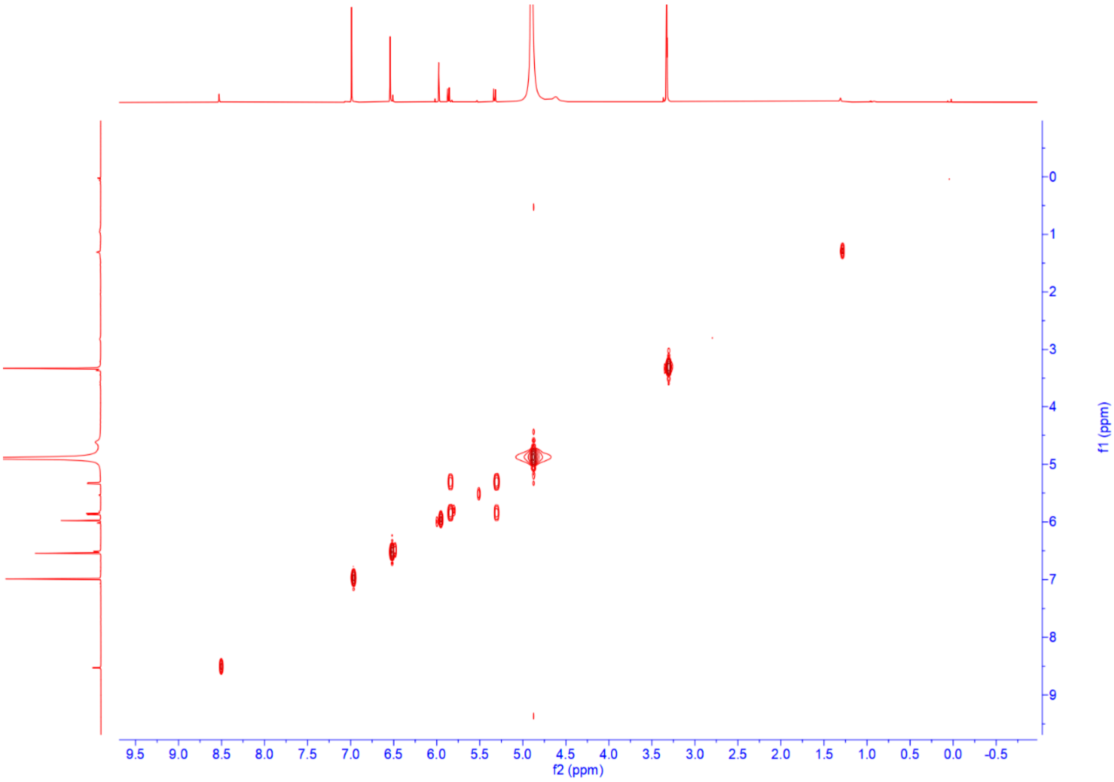

Figure S6. ^{1}H-^{1}H COSY spectrum of meichasu A (**1**).

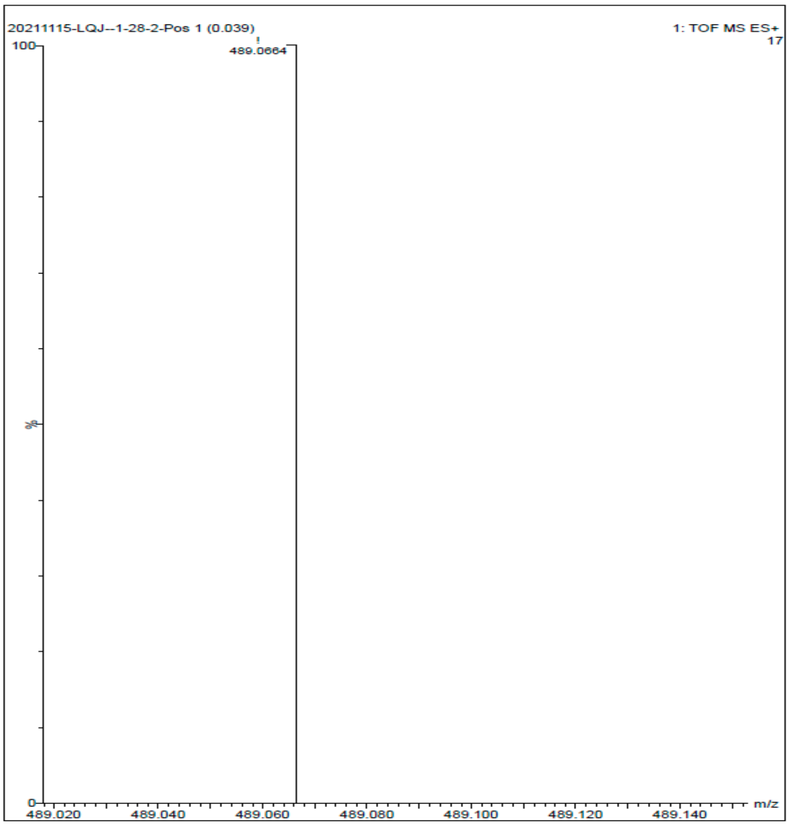

Figure S7. HR-ESIMS spectrum of meichasu A (**1**).

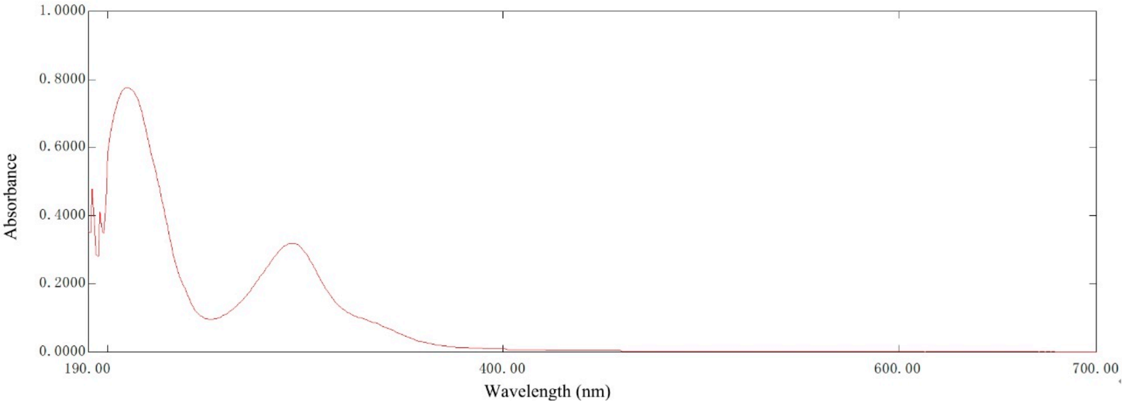

Figure S8. UV spectrum of meichasu A (**1**).

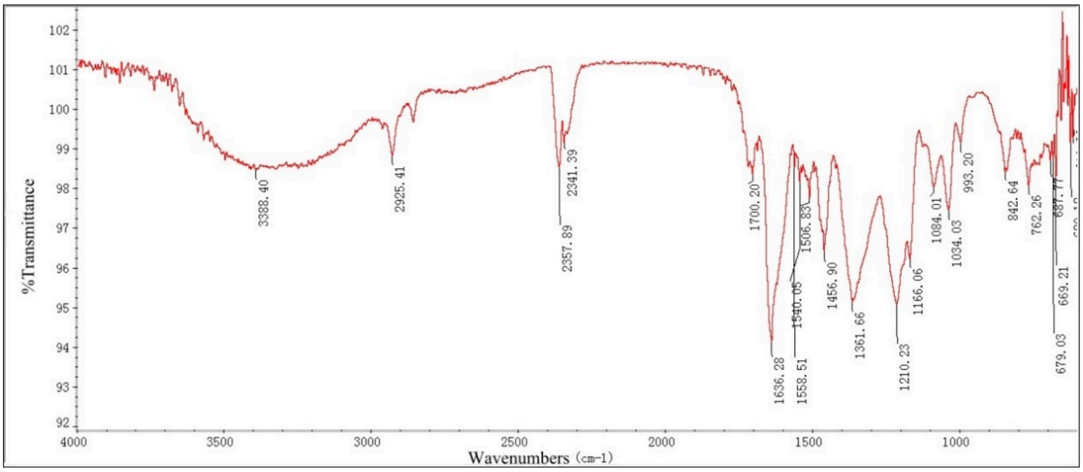

Figure S9. IR spectrum of meichasu A (**1**).

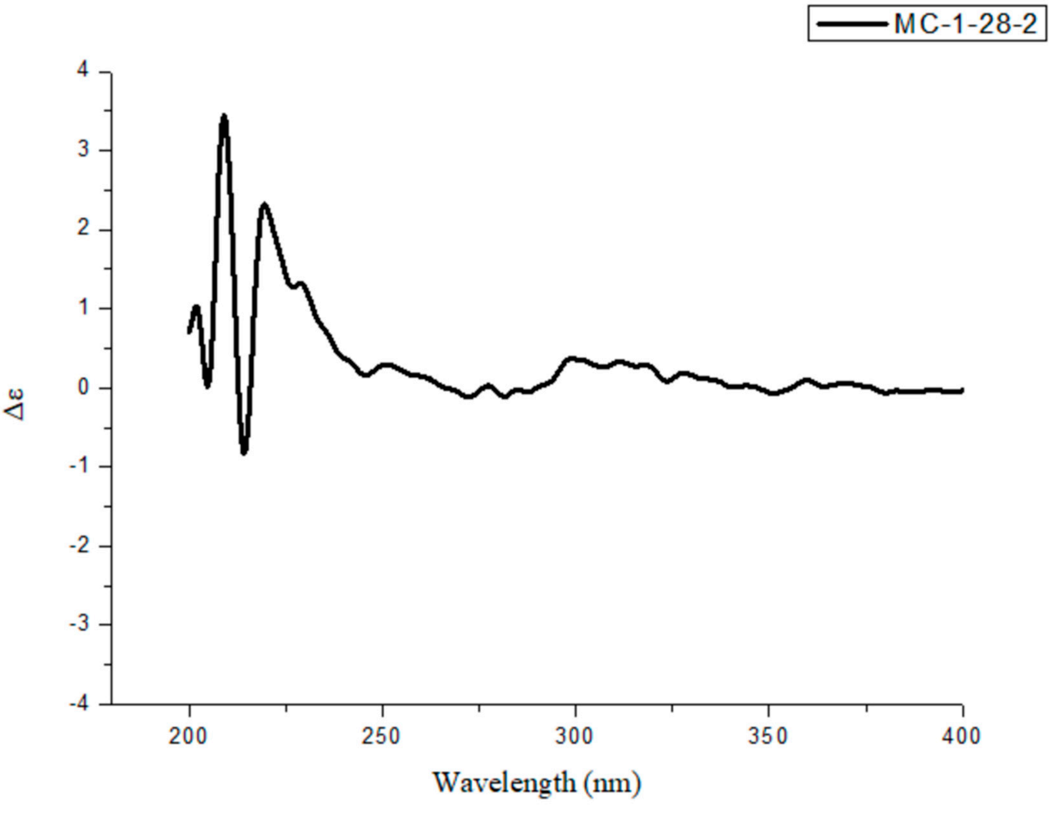

Figure S10. CD spectrum of meichasu A (**1**).

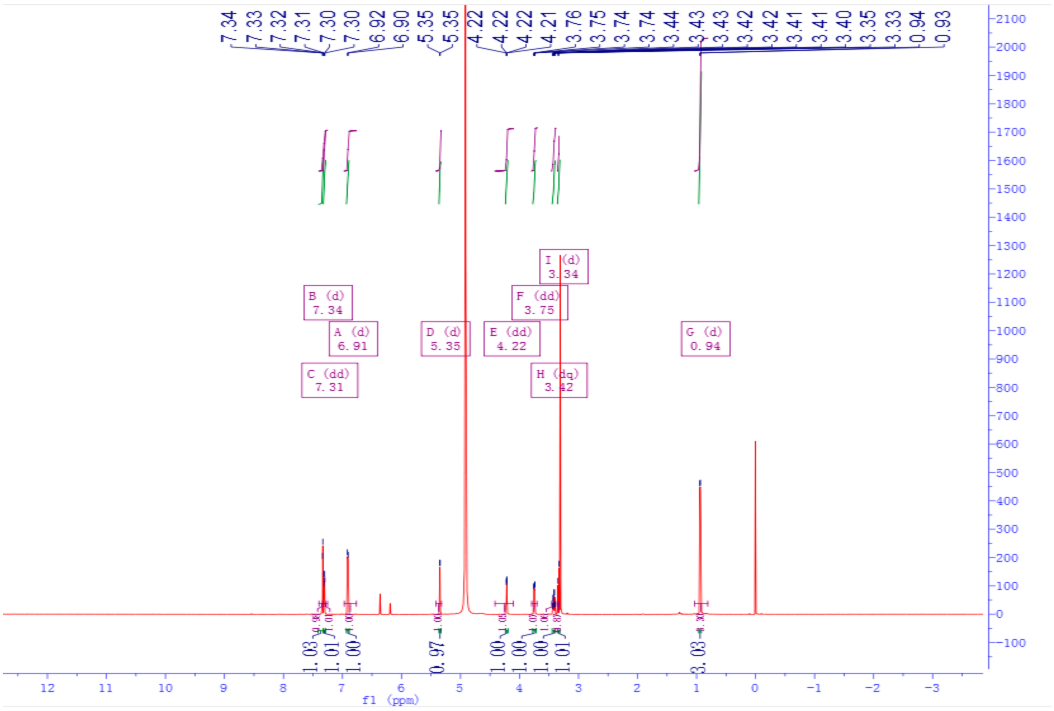

Figure S11. ^{1}H NMR spectrum of meichasu B (**2**) (CD_3OD, 600 MHz).

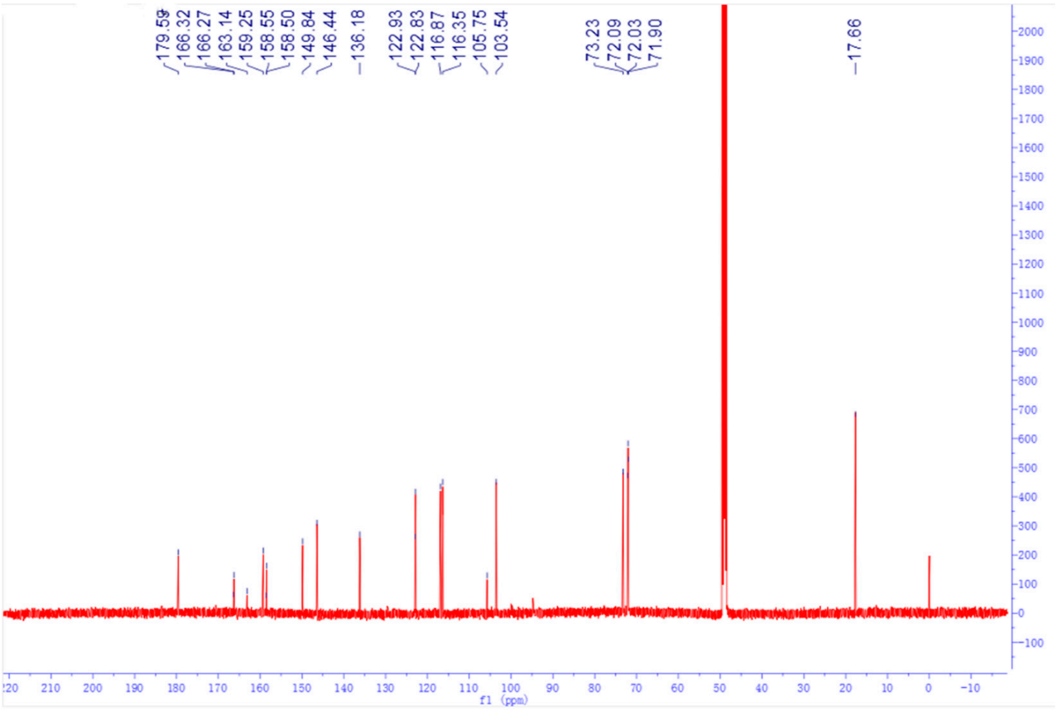

Figure S12. ^{13}C NMR spectrum of meichasu B (**2**) (CD_3OD, 151 MHz).

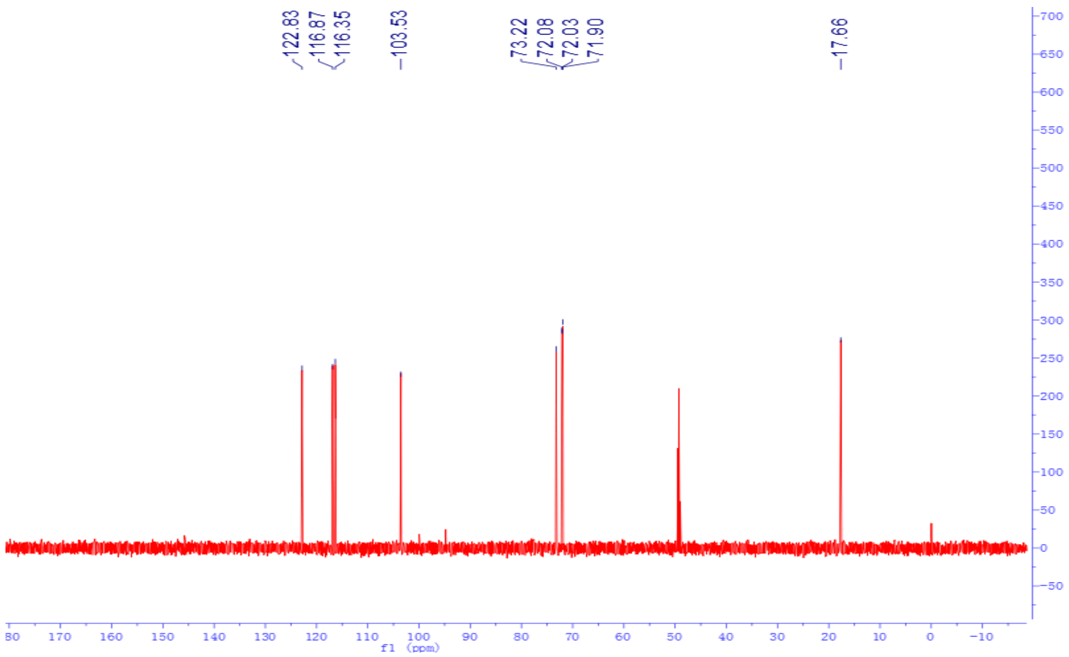

Figure S13. DEPT 135° spectrum of meichasu B (**2**) (CD_3OD, 151 MHz).

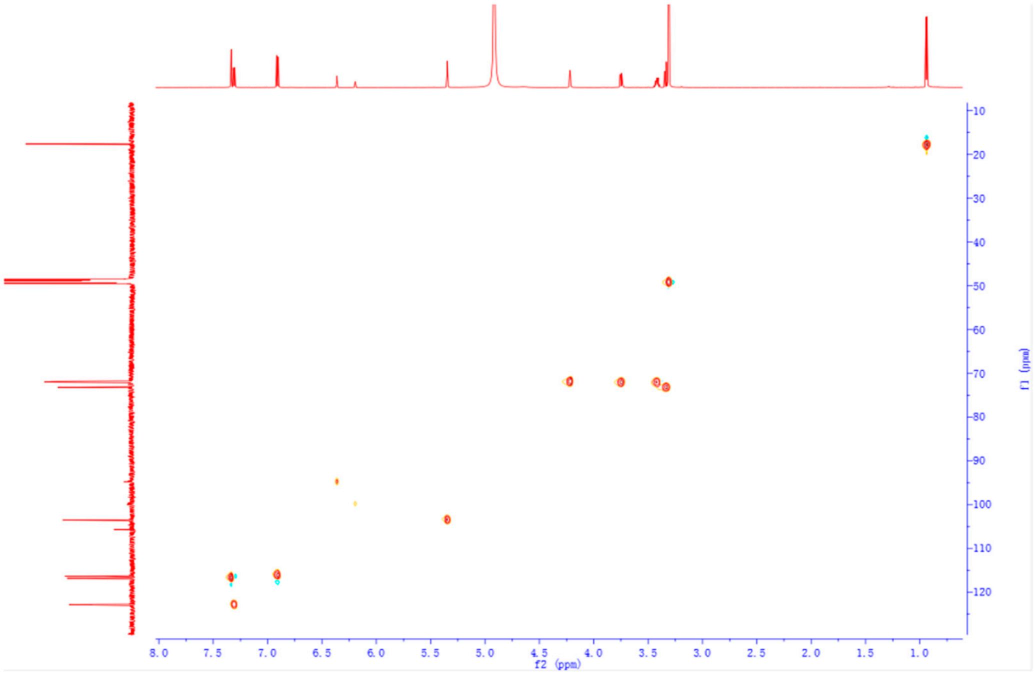

Figure S14. HSQC spectrum of meichasu B (**2**).

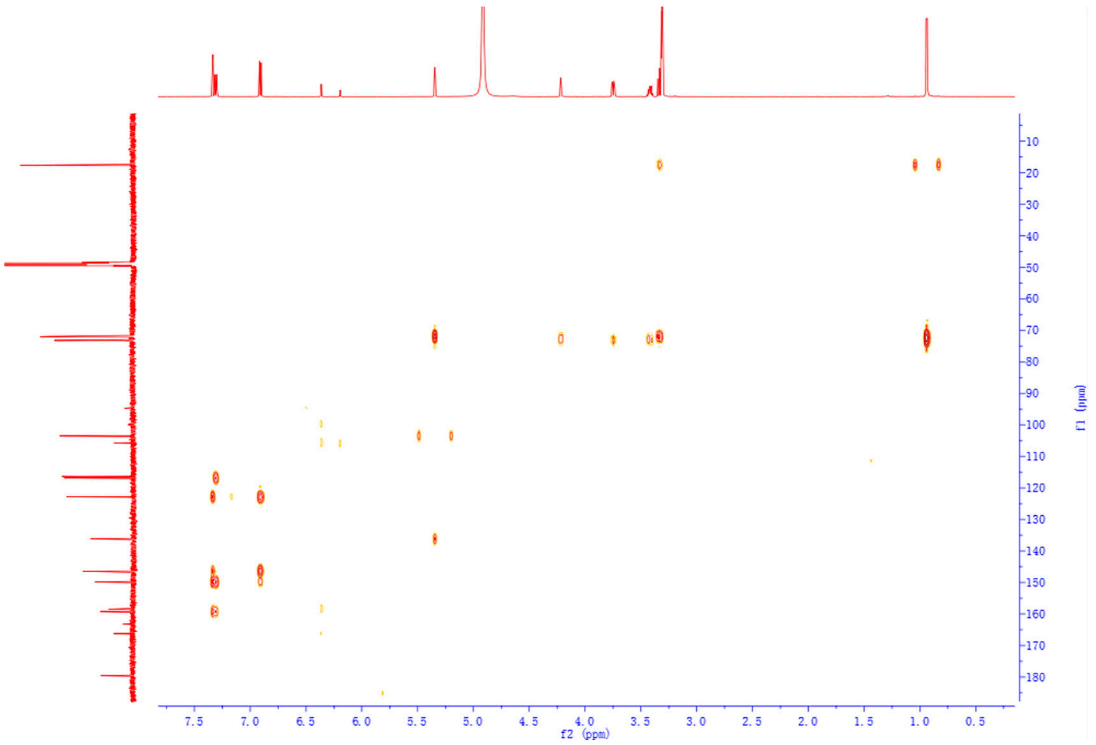

Figure S15. HMBC spectrum of meichasu B (**2**).

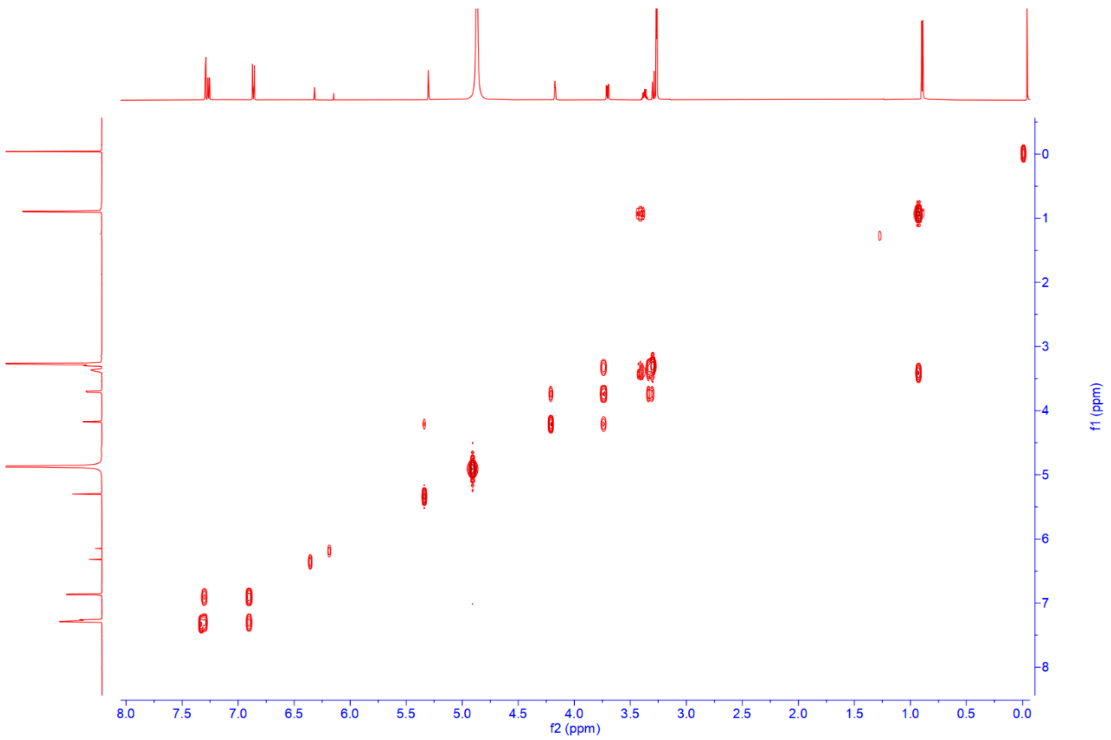

Figure S16. ^{1}H-^{1}H COSY spectrum of meichasu B (**2**).

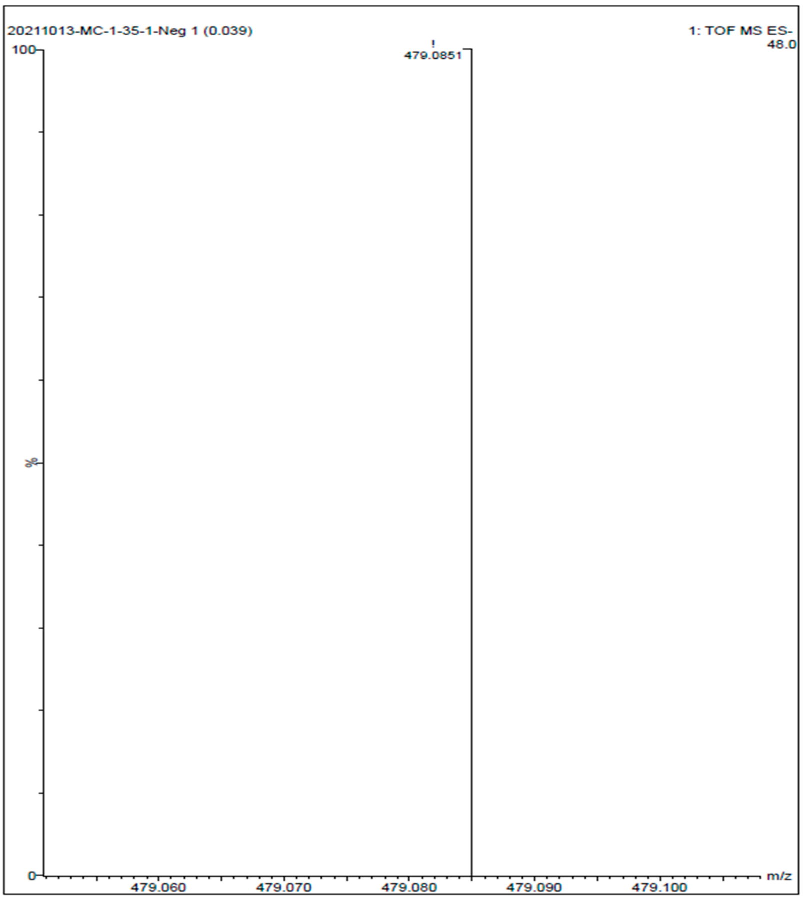

Figure S17. HR-ESIMS spectrum of meichasu B (**2**).

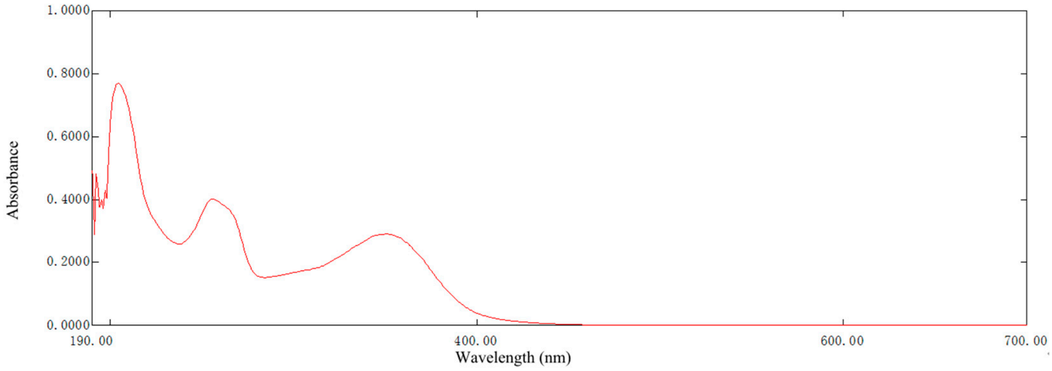

Figure S18. UV spectrum of meichasu B (**2**).

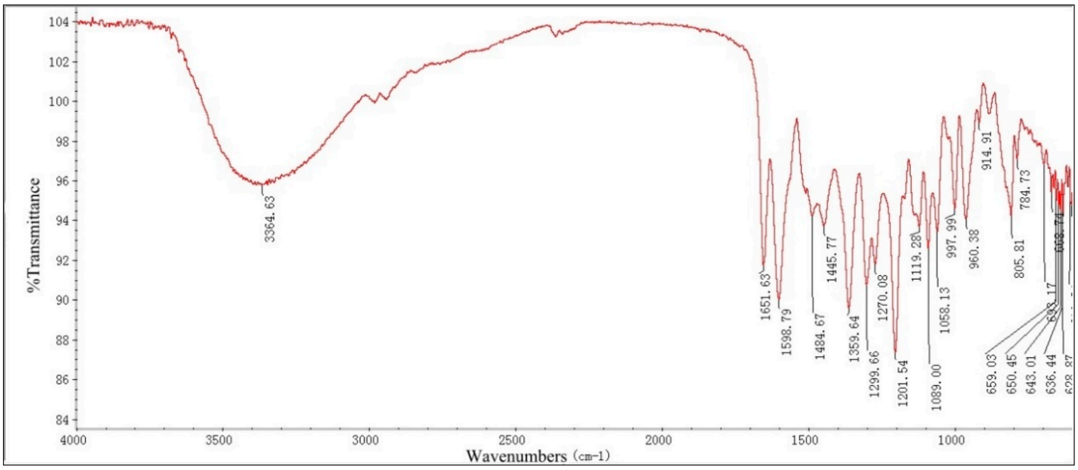

Figure S19. IR spectrum of meichasu B (**2**).
